# External flail chest stabilization; The simple, low-cost way

**DOI:** 10.34172/jcvtr.2020.58

**Published:** 2020-11-28

**Authors:** Efstratios Apostolakis, Nikolaos A Papakonstantinou, Alexandra Liakopoulou, Serafeim Chlapoutakis

**Affiliations:** ^1^Cardiothoracic Surgery Department, University Hospital of Ioannina, School of Medicine, Ioannina, Greece; ^2^Cardiac Surgery Department, Onassis Cardiac Surgery Center, Athens, Greece

**Keywords:** Thoracic Trauma, Flail Chest, External Stabilization, Respiratory Insufficiency

## Abstract

Flail chest is a life-threatening clinical entity which can be complicated by respiratory insufficiency. Paradoxical motion of a part of chest wall is the basic cause to put the blame on. Consequently, stabilization of the chest wall is occasionally of paramount importance to achieve early extubation in a patient with post-trauma respiratory insufficiency. Hereby, a simple, low cost, harmless and effective approach of external stabilization is presented.

## Introduction

Flail chest constitutes a serious complication of thoracic trauma.^1,2^ At least four rib fractures in two or more points of adjacent ribs are typical of flail chest.^3^ As a result, rigidness of chest wall is locally abolished, leading to paradoxical motion of the implicated part of chest wall,^3^ which, in combination with co-existing lung contusion, causes serious deterioration of patient’s respiratory function.^1-5^ In up to 30% of these patients, acute respiratory insufficiency, requiring mechanical ventilation and related to high mortality and morbidity rates, will be established.^1,4,5^ Unfortunately, mechanical ventilation does not prevent paradoxical motion of chest wall, so several stabilization techniques have been recommended, either via internal osteosynthesis of fractured ribs or even the sternum, or via external stabilization.^5^ Herein, we present a simple, cheap, though effective approach for external stabilization of the flail part of chest wall which was successfully applied in a trauma patient.

## Case Presentaion

**Figure 1:**
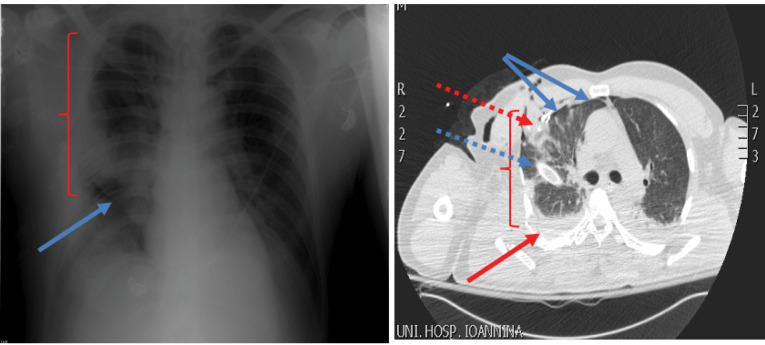
Chest x-ray and Computed Tomography on patient’s admission

**Figure 2:**
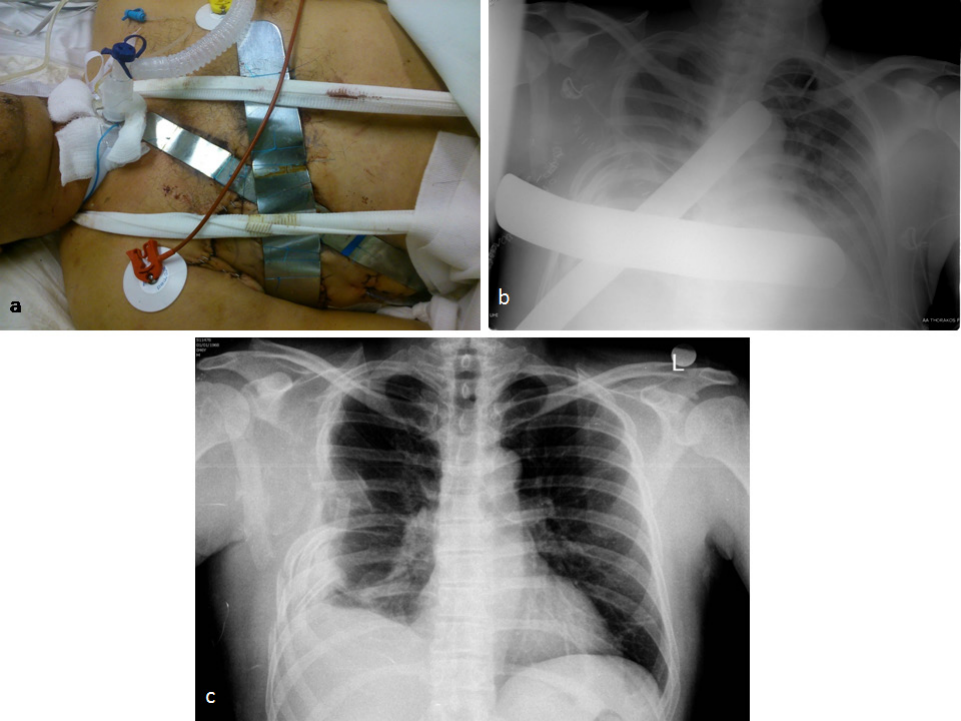
External stabilization with metallic bars

A 58-year-old patient, who was an excavator operator, was crashed down by the bucket of the excavator during work time. His injury resulted in right ribs comminuted fractures and in three infected penetrating traumas of his right anterolateral chest wall. The patient was transferred to hospital having hypoxia and air and blood leak from open wounds, though being hemodynamically stable (blood pressure=110/65 mm Hg, heart rate = 90 beats per minute (bpm)). Physical examination proved neither additional injuries nor any change in Glasgow Coma Scale. Chest x-ray showed right pneumothorax and hemothorax, severe pulmonary contusion of the right lung and multiple rib fractures from the second rib to the eighth one (Figure 1). Subsequently, a chest tube was placed in his right hemithorax. Arterial blood gases showed severe hypoxia (PO_2_ = 58 mm Hg, PCO_2_ = 31 mm Hg, SatO_2_ = 88%). The patient was intubated and transferred to the operating theatre, where the presence of rib fractures from the second to the eighth rib and of three lung rupture sites with massive air leak were noticed. Debridement of chest wall traumas, suturing of lung rupture sites with a continuous 4-0 Prolene suture through a right lateral thoracotomy and drainage of right pleural cavity via a wide chest tube were performed (Figure 1). During next postoperative week, the patient presented serious hypoxia and paradoxical motion of the anterolateral chest wall. Despite ventilatory support with FiO_2_ of 70%-80% and positive end expiratory pressure of 15 mm Hg, patient’s oxygenation was affected (PO_2_ = 62 mm Hg, PCO_2_ = 36 mm Hg, SatO_2_ = 90%) and his extubation was impossible. Consequently, stabilization of chest wall was decided on the seventh postoperative day. After patient’s sedation, two reusable malleable, metallic bars, 25 and 30 centimeters long were chosen and sutured crosswise, so as complete contact with the mobile part of the anterolateral chest wall to be achieved. Multiple non-ischemic, interrupted, 2-0 Nylon sutures were used suturing the bars on the patient’s skin and subcutaneous layer and between them. The edges of metallic bars were sutured on the adjacent normal, stable chest wall resulting in apparent limitation of paradoxical motion (Figure 2a). After chest wall stabilization, arterial blood gases were clearly improved. (PO_2_ = 75 mm Hg, PCO_2_ = 36 mm Hg, SatO_2_ = 96%) Respiratory function improved, hypoxia regressed and the patient was successfully extubated on the third post-stabilization day having satisfactory air blood gases and chest x-ray (Figure 2b). Lung contusion resolution along with mechanical stabilization of the chest were probably the contributors of this achievement. External metallic bars were removed on the ninth post-stabilization day, without any sign of skin necrosis, by simply dividing the stabilizing non-ischemic sutures. The patient was discharged on the 16th post-stabilization day in good health condition without paradoxical motion of his chest wall (Figure 2c). An informed consent was obtained from the patient for the publication of this data.

Chest x-ray on admission on the left and Computed Tomography imaging of patient’s thorax on the right after his first operation on the right showing pneumothorax (blue arrows) and hemothorax (red arrow) of his right hemithorax, severe pulmonary contusion (red dotted arrow) of his right lung, subcutaneous emphysema and multiple rib fractures from the second rib to the eighth one.(red bracket) A chest drainage tube is in place on the CT image (blue dotted arrow)

**a)** Two reusable, malleable, 25 and 30 centimeters long, metallic bars were sutured crosswise in complete contact with the mobile part of the anterolateral chest wall. The edges of metallic bars were sutured on the adjacent normal, stable chest wall; **b)** Improved chest x-ray on the third post-stabilization day; **c)** Chest x-ray on patient’s discharge)

## Discussion

The chest wall stabilization approach previously described, could be called “poor’s stabilization”. It has many advantages compared to an open stabilization approach. The abolition of potential “open rib reconstruction” is its main advantage. In addition, all open stabilization techniques described are high-cost, require full-anesthesia and therefore, they are related to all relevant complications. Our method constitutes a safe, low-cost and effective approach, that requires no anesthesia but only sedation. Indeed, current, established stabilization approaches (stainless steel reconstruction plates, intramedullary k-wires, Adkins Struts, Judet struts)^5^ have the following prerequisites: firstly, there should not be extended rib destruction, so as for the intact parts of the ribs to be nailed and secondly, trauma should not be infected. If the latter happens, the implanted stabilization system will be very quickly infected requiring its surgical removal. The concept of the hereby recommended stabilization approach is that it achieves the stabilization of the mobile part of chest wall on the rest, stable chest wall, thus preventing from paradoxical motion.

## Conclusion

Lack of complications and no need of general anesthesia make this technique particularly precious in the management of flail chest. Probably, lung contusion resolution along with mechanical stabilization are the main contributors for extubation achievement.

## Competing interests

None declared.

## Ethical issues

None.
